# Intraurethral co-transplantation of bone marrow mesenchymal stem cells and muscle-derived cells improves the urethral closure

**DOI:** 10.1186/s13287-018-0990-2

**Published:** 2018-09-21

**Authors:** Anna Burdzinska, Bartosz Dybowski, Weronika Zarychta-Wiśniewska, Agnieszka Kulesza, Marta Butrym, Radoslaw Zagozdzon, Agnieszka Graczyk-Jarzynka, Piotr Radziszewski, Zdzislaw Gajewski, Leszek Paczek

**Affiliations:** 1 Department of Immunology, Transplantology and Internal Diseases, Medical University of Warsaw, Nowogrodzka 59, 02-006 Warsaw, Poland; 2 Department of Urology, Medical University of Warsaw, Warsaw, Poland; 3 Division of Dermatology and Venereology, Department of Clinical Sciences, Lund University, Lund, Sweden; 4 Department of Clinical Immunology, Medical University of Warsaw, Warsaw, Poland; 5 Department of Bioinformatics, Institute of Biochemistry and Biophysics, Polish Academy of Sciences, Warsaw, Poland; 6 Department of Immunology, Medical University of Warsaw, Warsaw, Poland; 7 Department of Large Animal Diseases with Clinic, Veterinary Research Centre and Center for Biomedical Research, Faculty of Veterinary Medicine, Warsaw University of Life Sciences (WULS - SGGW), Warsaw, Poland

**Keywords:** Co-transplantation, Muscle-derived cells, Mesenchymal stem cells, Urinary incontinence, Autologous transplantation, Cell transplantation

## Abstract

**Background:**

Cell therapy constitutes an attractive alternative to treat stress urinary incontinence. Although promising results have been demonstrated in this field, the procedure requires further optimization. The most commonly proposed cell types for intraurethral injections are muscle derived cells (MDCs) and mesenchymal stem/stromal cell (MSCs). The aim of this study was to evaluate the effects of MDC-MSC co-transplantation into the urethra.

**Methods:**

Autologous transplantation of labeled MDCs, bone marrow MSCs or co-transplantation of MDC-MSC were performed in aged multiparous female goats (*n* = 6 in each group). The mean number of cells injected per animal was 29.6 × 10^6^(± 4.3 × 10^6^). PBS-injected animals constituted the control group (*n* = 5). Each animal underwent urethral pressure profile (UPP) measurements before and after the injection procedure. The maximal urethral closure pressure (MUCP) and functional area (FA) of UPPs were calculated. The urethras were collected at the 28th or the 84th day after transplantation. The marker fluorochrome (DID) was visualized and quantified using in vivo imaging system in whole explants. Myogenic differentiation of the graft was immunohistochemically evaluated.

**Results:**

The grafted cells were identified in all urethras collected at day 28 regardless of injected cell type. At this time point the strongest DID-derived signal (normalized to the number of injected cells) was noted in the co-transplanted group. There was a distinct decline in signal intensity between day 28 and day 84 in all types of transplantation. Both MSCs and MDCs contributed to striated muscle formation if transplanted directly to the external urethral sphincter. In the MSC group those events were rare. If cells were injected into the submucosal region they remained undifferentiated usually packed in clearly distinguishable depots. The mean increase in MUCP after transplantation in comparison to the pre-transplantation state in the MDC, MSC and MDC-MSC groups was 12.3% (± 11.2%, not significant (ns)), 8.2% (± 9.6%, ns) and 24.1% (± 3.1%, *p* = 0.02), respectively. The mean increase in FA after transplantation in the MDC, MSC and MDC-MSC groups amounted to 17.8% (± 15.4%, ns), 15.2% (± 12.9%, ns) and 17.8% (± 2.5%, *p* = 0.04), respectively.

**Conclusions:**

The results suggest that MDC-MSC co-transplantation provides a greater chance of improvement in urethral closure than transplantation of each population alone.

## Background

Cell therapy has been proposed as an alternative method to treat urinary incontinence. Since the first report on this topic in 2000 [1], many efforts have been undertaken to elaborate this procedure to the clinical grade. At present, although numerous promising results have been demonstrated, there is a general consensus that the procedure requires further optimization [2].

There are two cell types which are most commonly proposed for this purpose: muscle-derived cells (MDCs) and mesenchymal stem/stromal cell (MSCs) [3]. MDCs are either myoblasts or multipotent muscle-derived stem cells - both cell types possess unquestionable myogenic potential demonstrated in vitro and in vivo [4, 5]. The postulated mechanism of action in regard to MDC transplantation is the direct myogenic differentiation, which could support the strength of the impaired urethral sphincter. In cases of transfer of myoblasts into the skeletal muscle tissue, the key problem is the low survival rate after transplantation, even if performed as an autologous procedure [6, 7]. There are several potential causes of graft elimination from the site of injection - one of them is the local inflammatory response to cell delivery [7, 8]. 

In contrary to myoblasts, the myogenic potential of MSCs both in vitro and in vivo after intramuscular injection is very limited. The ability of isolated MSCs to participate in muscle regeneration is based on fusion rather than on true differentiation [9, 10]. However, MSCs possess well-documented high secretory activity and are believed to stimulate endogenous progenitor cells and angiogenesis by a paracrine mechanism [11]. Moreover, MSCs have gained increasing interest because of their immunomodulatory properties. Although the effect of MSCs on immune response is complex and not fully understood, there is a general agreement that this activity has strong anti-inflammatory and pro-regenerative components [12]. The MSC injection procedure has been tested as a concomitant treatment in solid organ transplantation [13], but also as a co-injection with other cell types i.e. cardiac or neural stem cells [14, 15]. The combination of skeletal myoblasts and bone marrow (BM)-derived MSCs as a therapeutic approach was proposed for the first time by Carvalho et al. [16]. The data from co-culture experiments published previously by our group suggest that myoblasts and MSCs can support each other in muscle regeneration [10]. Therefore, we hypothesized that myoblast-MSC co-transplantation could be more effective in the treatment of urethral sphincter deficiency than injection of myoblasts or MSCs alone. The aim of this study was to evaluate the effects of myoblast-MSC co-transplantation into the urethra.

## Methods

### Animals, study groups and the overall study schedule

**Fig. 1:**
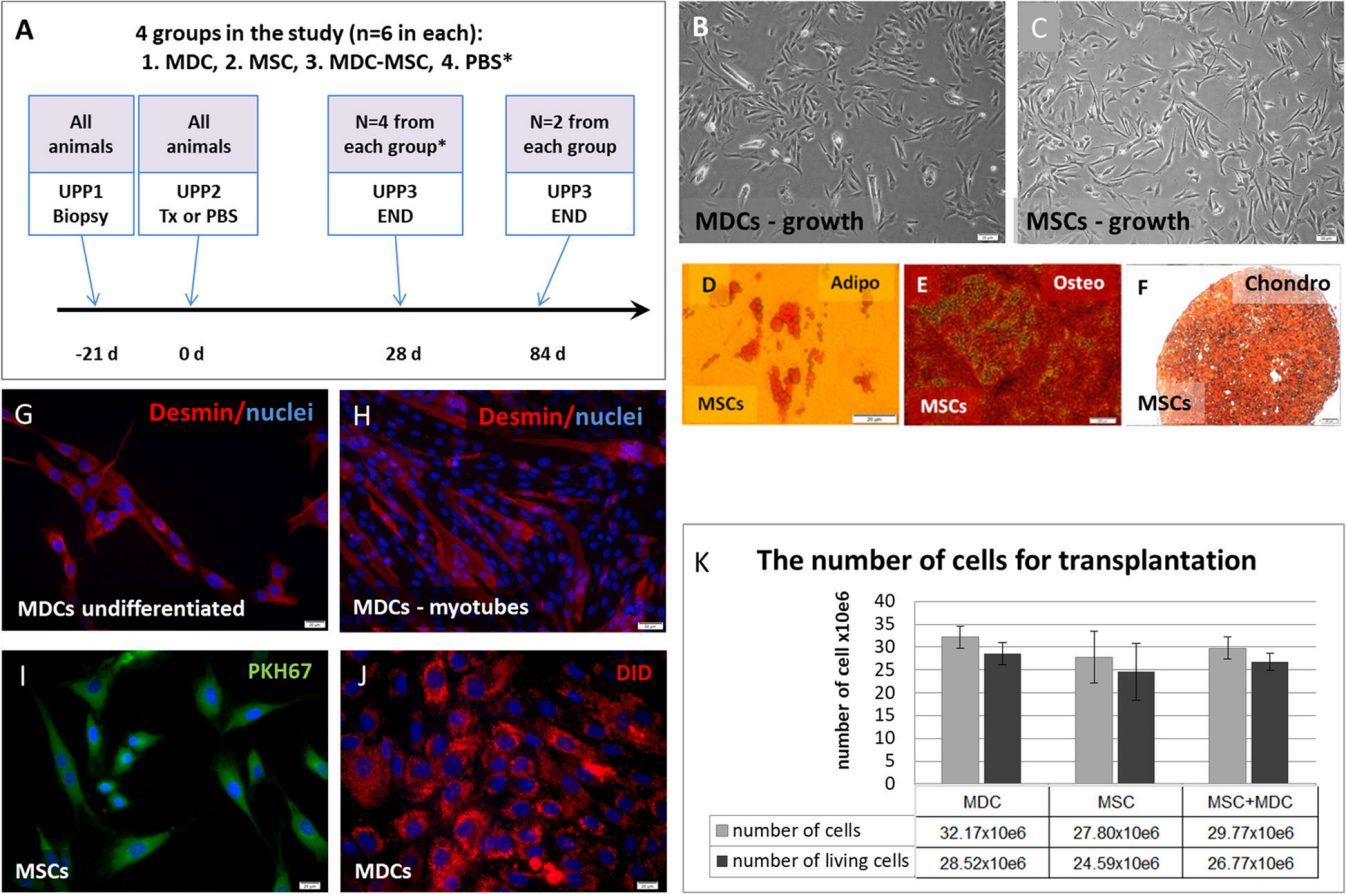
Study schedule and preparation of cells for transplantation. **a** Study schedule. UPP, urethral pressure profile; Tx, transplantation; * - in the PBS group one animal was lost during the experiment; **b** proliferating caprine muscle-derived cells (MDCs); **c** proliferating caprine bone marrow mesenchymal stem cells (MSCs); **d** MSCs differentiated into adipocytes - Oil Red O staining; **e** MSCs differentiated into osteocytes - Alizarin Red staining; **f** MSCs differentiated into chondrocytes - Safranin O staining; **g** undifferentiated MDCs immunostained for desmin (red fluorescence); **h** MDCs after myogenic differentiation - multinucleated myotubes can be observed (desmin is red and cell nuclei are blue); **i** MDCs labeled with PKH67 - a green fluorophore; **j** MDCs labeled with DID - a far red fluorophore; **k** mean (± SEM) number of cells (in total or only living cells) used for transplantation in the experimental groups. No statistical differences were noted between groups

The experiments were performed on adult female Polish white landrace goats. A total of 24 goats weighing about 40 kg was included in the study. All goats had undergone multiple deliveries and were more than 6 years old. No signs of lower urinary tract disease were observed in the experimental animals at the moment of inclusion to the study. The study schedule is presented in Fig. 1a.

All goats underwent general anesthesia three times during the experimental course. The first anesthesia was to take the muscle and BM biopsies and to measure the baseline urethral pressure profile (UPP). The cells were isolated from muscle tissue and BM and were propagated for 3 weeks in vitro. After 3 weeks, all animals were anesthetized again for the second baseline UPP measurement followed by autologous intraurethral PBS injection (control group, *n* = 6) or cell transplantation (3 experimental groups): (1) myoblasts - MDC group, *n* = 6, (2) mesenchymal stem cells - MSC group, *n* = 6 and (3) co-transplantation of myoblasts and MSCs - MDC-MSC group, *n* = 6). From each group, four animals ended the experiment at day 28 and two animals ended at day 84. On the last day of the experiment the goats underwent the third, final anesthesia. The UPP was measured and the goats were euthanized to collect the tissues for further analysis.

The study protocol and all procedures were reviewed and approved by the Local Ethics Animal Welfare Commission (permission number 39/2012). The study was designed to minimize the impact of the learning curve on the results. It was planned to conduct the experiments in six sets - in each set four animals should undergo the same procedures on the same day (one control and three experimental animals - one from each experimental group). Only minor deviations from this rule occurred and they will be described in the “Results” section. Additionally, the 1-month and 3-month sets were conducted alternately. The animals were assigned to the groups randomly.

### Urethral profilometry

For the urodynamic procedure, the animals were sedated by intramuscular (i.m.) administration of midazolam 0.4 mg/kg/body weight (b.w.) (Midanium®, Polfa Warszawa S.A.) followed by intravenous (i.v.) administration of propofol in bolus (2–4 mg/kg b.w. depending on the reaction) (Propofol, Scanofol®, ScanVet). The static UPP was determined on animals placed in sternal recumbency. The measurements were carried out during recovery from propofol action in a state of light anesthesia (palpebral reflex present) as our group has previously shown that this kind of anesthesia only moderately affects the UPP measurements [17]. A twin-lumen catheter (6Fr, Laborie) with side holes was withdrawn from the bladder and the urethra at a constant speed of 2.5 mm/s. Channels were perfused with sterile warm water at a rate of 0.6 ml/min. External pressure transducers (TSD104A, Biopac Systems) were localized at the level of the bladder and calibrated at atmospheric pressure. A Biopac MP100 unit (Biopac Systems) with Acqknowledge 3.9.1 software were used for data acquisition and analysis. The system was calibrated on each experimental day. At least three UPP records were obtained during one session in each animal. The selection of data for further evaluation was two-step. First, only profiles without distinct artifacts were chosen. Second, in each animal/time point the profile with the highest values (assumed least affected by anesthesia) was chosen for analysis. The calculated UPP parameters were the maximal urethral closure pressure (MUCP; the difference between maximal urethral pressure and the pressure in the urinary bladder) and the functional area (FA; the area under the urethral-closure pressure curve).

### Bone marrow (BM) and skeletal muscle biopsies

The biopsy procedures were performed after completing UPP recording. The animals were deeply sedated with an i.v. bolus of propofol, and intubated. Then, the anesthesia was maintained with isoflurane (2%, Aerrane, Baxter Polska). BM aspirate was aseptically collected from a radial bone to a probe with 500 U of heparin. A skeletal muscle sample was taken from vastus medialis muscle and placed in sterile PBS (Invitrogen) supplemented with 1% of penicillin-streptomycin (Life Technologies) solution. Penicillin with dihydrostreptomycin; Shotapen Virbac®; 1 ml/10 kg/b.w., i.m. was used for antibiotic prophylaxis. During the procedure, analgesia was achieved by i.v. injection of fentanyl (Fentanyl WZF, Polfa). After the procedure, a non-steroidal anti-inflammatory drug - tolfenamic acid (Tolfine®, Vetoquinol Polska, 2 mg/kg/b.w., i.m.) was administrated.

### Cell isolation, identification and culture for transplantation

The protocols for caprine myoblast and BM-MSC isolation have previously been described in detail by our group [18]. Briefly, after mechanical disassociation, washing and centrifugation the BM samples were suspended in medium composed of Dulbecco’s modified Eagle’s medium with low glucose (DMEM-LG; Sigma Aldrich) supplemented with fetal calf serum (FCS; 10%; Life Technologies) and antibiotic–antimycotic solution (penicillin-streptomycin-amphotericin B; 1.5%; Life Technologies) and seeded on a plastic surface. After 4 days the medium with non-adherent cells was removed and first colonies of fibroblast-like adherent cells could be observed. Myoblasts were isolated by mechanical dissociation of muscle tissue followed by enzymatic digestion (0.15% (w/v) protease, Sigma Aldrich, P8811) in DMEM, (37 °C, 60 min). Pre-plating was performed 1 h after seeding. MDCs were grown on BD Primaria™ culture dishes in DMEM supplemented with 10% FCS, 5% horse serum (HS; Life Technologies) and 1.5% antibiotic-antimycotic mixture. The cells were passaged before reaching the critical level of confluence in which myoblasts underwent spontaneous myogenic differentiation. The identification procedures encompassed testing the differentiation capability: myogenic for muscle-derived cells and osteogenic, adipogenic and chondrogenic for BM-derived cells, followed by the appropriate staining procedure. Additionally, the identity of MDCs was verified by immunocytochemical staining for desmin (mouse anti-desmin 1:30, clone D33, Dako, overnight, 4 °C, followed by secondary Alexa Fluor 584- conjugated Donkey Anti- Mouse antibody, 1:100, Jackson ImmunoResearch, 1 h, room temperature (RT)) and BM cells were tested for colony-forming ability. The cells isolated from all animals in one experimental set were expanded in parallel in order to achieve a sufficient number of cells for transplantation on the same day for each animal (in each set one animal was transplanted with MDCs, one with MSCs and one co-transplanted with MDC-MSCs to avoid day-dependent variations).

### Cell preparation for transplantation

On the day of transplantation, cells were detached from culture dishes and counted. The desired number was 30 million cells per animal. Cells were labeled with fluorescent membrane fluorophores prior to transplantation. Optimally, 40 million cells from one animal were designated for staining (to have a reserve, because the preparation procedure caused a significant lowering of cell number). For co-transplantation, 20 million MDCs and 20 million MSCs constituted the desired amount. If fewer cells were collected, all were labeled. In the MDC and MSC groups, cells were stained with far red dye - DiLC18(5)-DS 1,1′-Dioctadecyl-3,3,3,-tetrametlylindodicarbocyanine-5,5-disulfonic acid( DID), excitation wavelength (Ex) = 650 nm, emission wavelength (Em) = 670 nm (AAT Bioquest). In the co-transplanted group, MDCs were labeled with DID, and MSCs were labeled with a green dye - PKH67 (Ex = 490 nm, Em = 502 nm, Sigma). Cells were washed twice before staining. Then, cells were incubated either with DID (10 μM in PBS, 15 min at 37 °C) or with PKH67 (5 μM PKH67 in Diluent C provided by the manufacturer, 4 min at RT). After staining, cells were washed twice and suspended in PBS to achieve a total volume of 400 μl per animal (in the MDC-MSC group - 200 μl per population). Then, cells were counted again with evaluation of cell viability (Trypan blue staining). At that point, MDC and MSC were combined in the co-transplantation group and became one sample. A 400-μl PBS probe was additionally prepared for control animals. The cells were transported to the surgery hall in a cooling box (~ 6 °C).

### Cell injection procedure

The cells were injected in a minimally invasive way from the lumen of the urethra under endoscopic control. The procedure was performed under general anesthesia after recording of UPPs. To make the injections more accurate and more reproducible, we have used a prototype of a device which was precisely described in our recent paper [19]. This device is a metal tubule suitable for an intraurethral insertion, which stabilizes the urethra during injection and acts as a guide for an endoscopic needle. Before the injection, the urethral length of the animal was calculated from the UPP. The needle guide was placed in the urethral lumen in the appropriate location. The desired position was to achieve a delivery point about 2 cm from external urethral orifice (if the urethra was very short or very long the position was adjusted). Injections were performed with use of a cysto-urethroscope (8/9.8 Fr., Richard Wolf GmbH). The injection cannula (Ø 1.0/0.6 mm, 300 mm length, Richard Wolf GmbH, Germany) was equipped with a needle lock for limiting needle protrusion. The cysto-urethroscope was connected to the imaging system (IMAGE,1 HUB, HD, H3-Z, Karl Storz).

For cell administration we used an automated system, which allowed for delivery of depots with the programmed speed and volume. After uploading the cell suspension to the system, the first drop coming from the needle tip was collected for microbiological tests (aerobic bacteria and yeast-like fungi). In each of 23 animals, 8 depots (cell suspension or PBS, 50 μl per depot) were administered with a constant speed of 11 μl/s. After administration of each depot, the needle was kept in the tissue for 1 min before slow retraction in order to improve local distribution of the fluid and to diminish cell suspension leakage from the site of injection. Four injections at 3, 5, 7 and 9 o’clock were carried out 2 cm from the external urethral sphincter and the same four positions were chosen more distally so in theory depots were placed in two planes. The procedure was performed in all animals by one urologist. The urologist who performed urodynamic measurements and injection procedures was blinded to the assignment of animals to particular groups.

### Tissue collection and procedure

At the end time point, the final UPP was recorded using the anesthetic regimen as described before. Then, the animals were euthanized and whole urethras with urinary bladders were collected. The tissues were fixed in 4% (w/v) *p*-formaldehyde in PBS, pH = 7.25 for 1–3 h. Next, the samples were rinsed for three consecutive days in PBS (once per day), and then immersed in 18% (w/v) sucrose in which the material was stored at 4 °C until analyzed using the IVIS® Spectrum imaging system, and finally frozen at a temperature of − 80 °C.

### Ex vivo imaging of urethras

We have exploited the in vivo imagining system (IVIS® Spectrum) for screening of explanted urethras in order to search for transplanted cell depots. The procedure for ex vivo imagining was recently optimized and described in detail by our group [20]. We have demonstrated that under certain conditions it is a suitable method for a preliminary assessment of transplanted cell survival and location in the entire isolated organ. Briefly, the urethras were manually cut into fragments of about 0.5 cm in length. As the average female caprine urethra is about 2.5–4.0 cm long, it resulted in 5–7 fragments. Prepared urethral fragments were laid on a Petri dish, always in the same arrangement: the caudal side of the fragment on the top and the dorsal side of the urethra upwards. To visualize DID fluorochrome, ex vivo imaging was performed with the IVIS® Spectrum system (Caliper Life Sciences) using Ex of 640 nm and Em of 680, 700, 720, 740, 760 and 780 nm, successively. First, an automated spectral separation algorithm (referred to as “spectral unmixing*”*) proposed by the manufacturer was used (Living Image 4.4. software, Caliper Life Sciences). This kind of analysis should separate the specific fluorescence signal originating from a particular fluorochrome and the background signal originating from the auto-fluorescence and food. The obtained images were additionally prepared for visualization by manual settings. The same ranges of minimum and maximum fluorescence strength were chosen for all analyzed records. The ranges were selected on the basis of PBS-injected urethras guided by the principle that in the control urethras the specific fluorescence is not visible. Additionally, a trial to estimate the intensity of DID-derived signal was undertaken. The fluorescence-specific signal was shown as the radiant efficiency:

Emission light (photons/s/cm^2^/sr)/Excitation light (μW/cm^2^).

The fluorescence signal for each urethra was counted by selecting the region of interest (ROI) and quantifying as total radiant efficiency:

TRE (photons/sec)/(μW/cm^2^).

TRE represents the sum of fluorescent pixels within the ROI. For each urethra six equal ROIs were chosen in such a way that ROIs encompassed the majority of the area of the urethral fragments including all visible spots after spectral unmixing. The TRE value was measured in each ROI. To cut off the unspecific signal, we performed the same measurements in all control urethras (counting of TRE from six equal ROIs). A mean TRE value was calculated from all 30 ROIs analyzed in control fragments (5 urethras by 6 ROIs in each). This mean value was used for normalization of the results obtained in the experimental groups. As the number of DID-stained cells differed between animals, the normalized TRE values obtained were adjusted by the appropriate coefficient (taking the amount of injected DID-stained cells into account). The green PKH67 dye was not visualized with this method because tissue auto-fluorescence would dramatically decrease the reliability of the data.

### Microscopic evaluation

Fragments in which specific signal was visible in IVIS® analysis underwent further procedures. Cross-sections of about 10 μm thickness were prepared using cryostat (MICROM HM 525, Microm). Specimens in which specific fluorescence was confirmed in raw preparations were immunohistochemically stained. The aims of this analysis were twofold: (1) to determine if transplanted cells differentiated into muscle; (2) to assess if this differentiation depends on the cell type and the location of the depot (within or outside the urethral sphincter). In order to achieve the aforementioned objectives, the preselected cross-sections were stained for desmin (the pan-myogenic marker) and sarcomeric myosin (the striated muscle marker). The specimens were fixed in acetone (6 min, − 20 °C), and blocked with 1% (w/v) bovine serum albumin (Sigma-Aldrich) + 5% (v/v) Normal Donkey Serum (Sigma-Aldrich) in PBS for 30 min at RT. They were incubated with the primary antibodies diluted in blocking solution for 90 min at RT. Anti-desmin antibody (Monoclonal Mouse Anti-Human Desmin, 1:30, DakoCytomation) or anti-myosin antibody (clone MF 20, 1:30, Developmental Studies Hybridoma Bank) were applied. After washing, samples were probed with a secondary antibody conjugated with Alexa Fluor 488 (Ex = 493 nm, Em = 519 nm; Jackson ImmunoResearch) diluted 1:100, for 60 min at RT. Specimens were washed again and nuclei were stained with 4',6-diamidino-2-phenylindole (DAPI). Imaging and analysis was performed with a fluorescence microscope Olympus IX51 and CellSens™ microscope imaging software or with the slide scanner Axio Scan.Z1 (Zeiss) and Zen Blue second edition software (Zeiss).

### Statistical analysis

STATISTICA software (StatSoft®) was used for data analysis. For most analysis non-parametric tests were used because of the limited number of data in the groups and/or abnormal data distribution based on the Shapiro-Wilk test. If relative data were compared (before versus after transplantation urodynamic measurements), the Wilcoxon matched-pairs signed-ranks test was used. If unrelated data from two groups were analyzed (i.e. comparison of normalized TRE values between time points within one experimental group), the Mann-Whitney test was used. For multiple groups with unrelated data (i.e. comparison of injected cell number between in three experimental groups), Kruskal-Wallis analysis of variance (ANOVA) was used. A *p* value less than 0.05 was considered as statistically significant. The urodynamic data were additionally analyzed to search for associations between different aspects of functional changes after transplantation. Pearson *r* correlation was used to measure the strength and direction of those associations. Those results were presented in scatter plots with trend lines and *r* values.

## Results

### The identity of isolated populations

The cell isolation was successful in all experimental animals within this study. Isolated caprine bone marrow (BM)-derived and muscle-derived cells displayed classical spindle-shaped morphology from the first days of culture, which did not change markedly until the time of transplantation (Fig. 1b, c). Both populations formed colonies while seeded at low density. The mean population doubling time of both cell types cultured in defined conditions did not differ significantly. To determine the identity of the isolated caprine BM-derived population, the cells were induced into multilineage differentiation. Specific staining confirmed the ability of caprine MSC to differentiate into adipocytes, osteocytes and chondrocytes (Fig. 1d–f). At the same time, MSCs were not able to gain a myogenic phenotype in monoculture. In contrast to MSCs, the majority of undifferentiated MDCs expressed desmin (Fig. 1g). Moreover, MDCs displayed the capability to fuse into multinucleated myotubes (Fig. 1h).

### Staining efficacy, cell number and cell viability data

At the day of transplantation (21st day of culture), in 6/18 cases, the number of collected cells was lower than the desired 40 million per animal, but only in 2 cases was this number lower than 30 million per animal. The staining procedure (including four washes) caused a significant loss of cells. The mean decline in cell number amounted to 26.5% of the initial population designated for staining. The short-term effectiveness of labeling was close to 100% regardless of the dye used (Fig. 1i, j). The mean (±SD) number of cells injected per animal was 29.6 × 10^6^ (± 4.3 × 10^6^). In particular experimental groups, the mean number of cells transplanted per animal amounted to 32.1 × 10^6^ (± 2.4 × 10^6^), 27.8 × 10^6^ (± 5.6 × 10^6^) and 29.7 × 10^6^ (± 2.3 × 10^6^) in the MDC, MSC and MDC-MSC groups, respectively. The mean viability of cells evaluated after staining did not differ distinctly between groups and came to 88.6% (± 2.5%), 88.1% (± 7.3%) and 90.1% (±5.1%) in the MDC, MSC and MDC-MSC groups, respectively (Fig. 1k). There were no significant differences between experimental groups in regard to either the injected cell number or their viability. The mean final ratio of MDC/MSC cells in the co-transplantation group was 1.17.

### The side effects, deviations from study schedule, microbiological tests

A total of 23 animals completed the study. One goat (from the PBS group) was lost during the experimental course because of respiratory depression and apnea during anesthesia induced with propofol. One goat appeared to be pregnant and was replaced by another animal. Therefore, two experimental sets (1st and 2nd) ended without PBS animals and in the 6th set there were two goats injected with PBS. One goat from the MDC-MSC group was moved from the 28-day group to the 84-day group, because of respiratory problems during anesthesia at day 28 and inability to gain a reliable urethral profile for analysis. Therefore, the MDC-MSC group ended with three (instead of four) animals in the 28-day group and three (instead of two) animals in the 84-day group. The microbiological examination of cellular suspensions (collected from the needle tip just before injection) revealed contamination of one sample (goat MDC-5 28d).

### The DID-stained cell survival in urethras at days 28 and 84

**Fig. 2:**
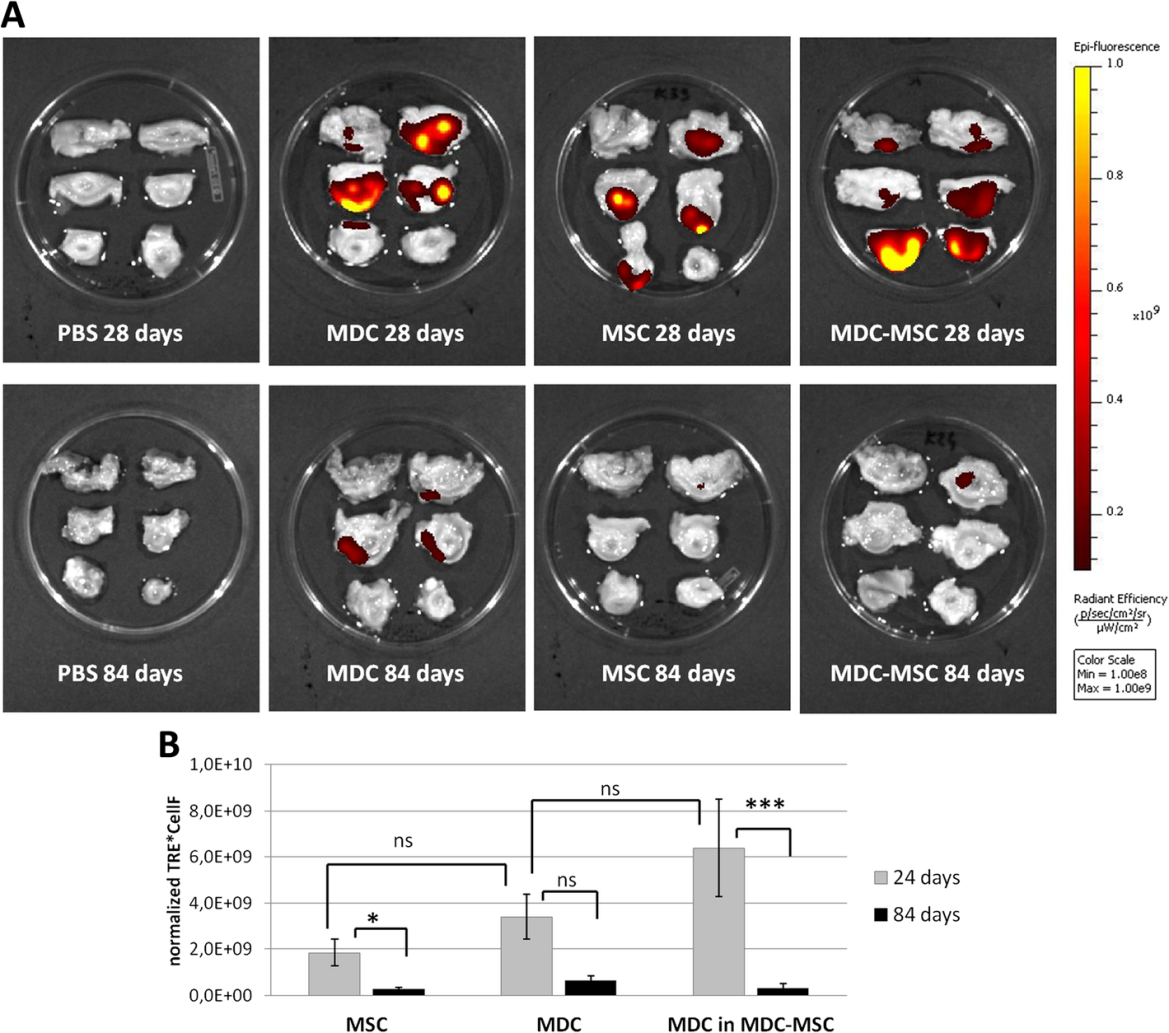
Visualization of 1,1′-dioctadecyl-3,3,3,-tetrametlylindodicarbocyanine-5,5-disulfonic acid (DID)-labeled cell depots in whole urethras using in vivo imaging system. **a** Each image represents one urethra cut into fragments. The yellow spots illustrate the areas with the strongest DID-derived fluorescence. The darker the spot, the weaker the signal. The specific fluorescence was identified using an automated spectral separation algorithm. The images are presented after manual alignment to the same, fixed ranges. The color scale bar shows the range of strongest to weakest signal (1 × 10^8^- 1 × 10^9^). The upper row presents urethras collected 28 days after either PBS injection (negative control) or transplantation of different cells types - muscle derived cells (MDCs), mesenchymal stem cells (MSCs) or MDC-MSC co-transplantation. The lower row presents urethras collected 84 days after injection. The best samples from each group were selected for this figure. **b** Intensity of DID-derived fluorescence in urethras from different experimental groups and different time points. The data are presented as mean (+/− SEM) total radiant efficiency (TRE, (photons/s)/(μW/cm^2^)) normalized to control, PBS-injected samples and adjusted to the number of injected DID-labeled cells. *p < 0.05; ***p < 0.001; ns, not statistically significant

The visualization of DID-derived fluorescence with the IVIS® imager revealed the presence of grafted cells in all transplanted urethras collected at day 28 regardless of experimental group. In urethras collected 84 days after transplantation, the spots were recognized in 6/7 urethras (no spots in one urethra from the MDC-MSC group). The visual evaluation of IVIS images with aligned min-max ranges suggested that the signal in urethras from the 84-day group was much weaker than in urethras from 28-day group (Fig. 2a). This observation was confirmed by the comparison of normalized TRE (nTRE) values adjusted to the number of injected DID-stained cells (cell number factor, CellF). The decline in the median signal between day 28 and day 84 was distinct in all types of transplantation. In the MSC and MDC-MSC groups the difference was statistically significant (*p* = 0.018 and *p* = 0.0007, respectively using the Mann Whitney U test, Fig. 2b). In the MDC group the decrease in signal between day 24 and day 84 was less pronounced and was not reach statistically significant (*p* = 0.15). We also compared this parameter between different types of transplantation within the same time point. The calculated median nTRE x CellF in 28-day urethras was the highest in the co-transplantation group and amounted to 6.4E + 09, whereas in the MSC and MDC groups it was 1.8E + 09 and 3.4E + 09, respectively. The differences between groups measured with Kruskal-Wallis ANOVA were not statistically significant. In 84-day urethras, the highest median signal was in the MDC group. The mean nTRE x CellF amounted to 2.7E + 08, 6.5E + 08 and 3.3E + 08 in the MSC, MDC and MDC-MSC groups, respectively. The differences between groups within 84-day urethras were also not statistically significant (Fig. 2b).

### The effect of cell transplantation on UPP parameters

**Fig. 3:**
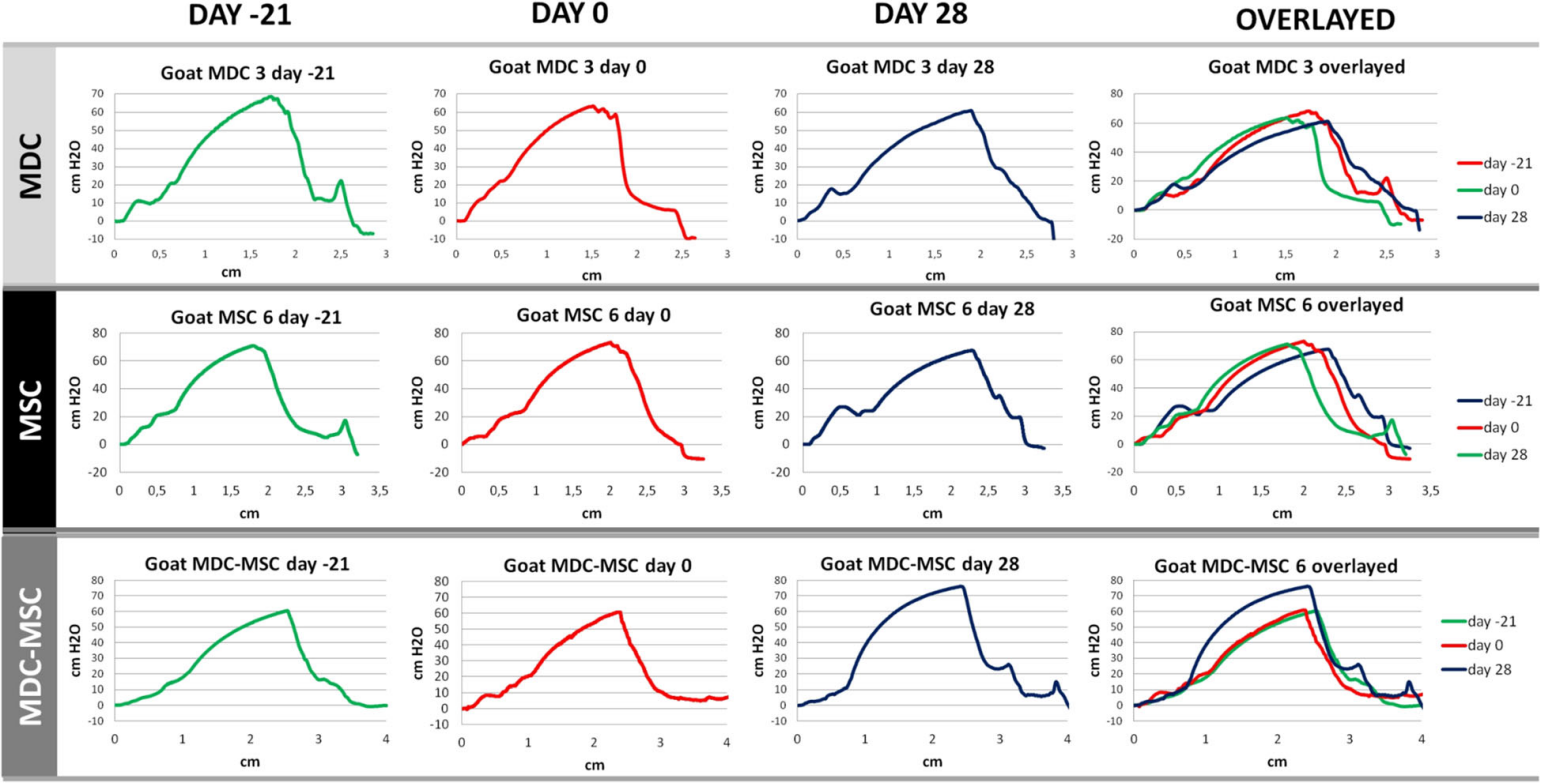
Urethral profile pressures (UPPs). The x-axis of graphs represents the length of the urethra (centimeters) and the y-axis represents the pressure recorded in the analyzed urethra (centimeters of H_2_O). Each row represents profiles recorded in one animal at different time points during the study schedule (before biopsy - day 21, before transplantation - day 0 and day 28 - 28 days after transplantation). Right column presents an overlay of all three UPPs from one row. Each row presents profiles of representative goats from the muscle-derived cells (MDC), mesenchymal stem/stromal cells (MSC) and co-transplanted group (MDC-MSC)

**Fig. 4:**
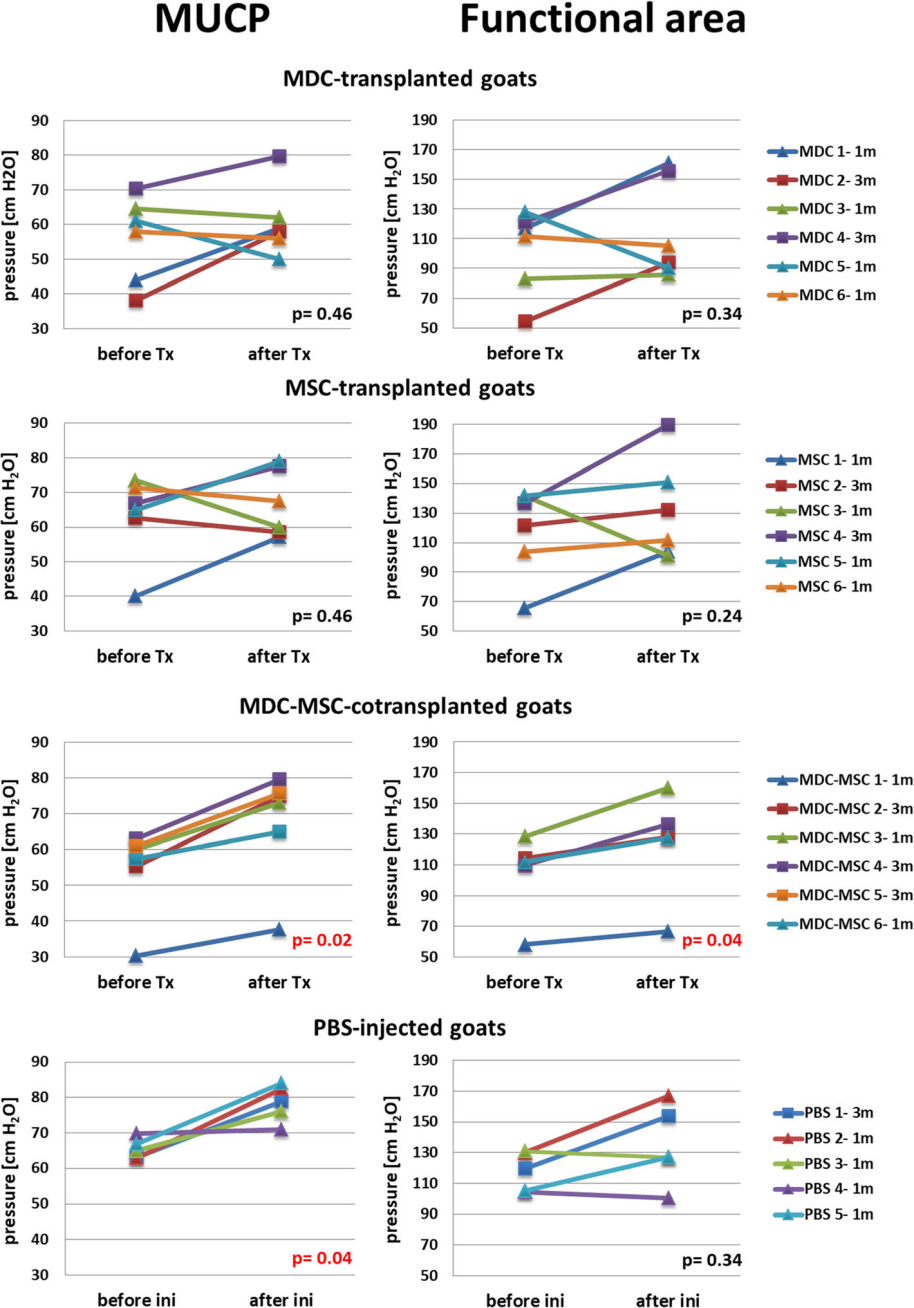
Functional response to cell transplantation or PBS injection in individual animals based on urethral profilometry. The left column shows the changes in maximal urethral pressure close (MUCP) and the right column shows the changes in the area under the profile curve (functional area - FA). “Before Tx/ini” values constitute the mean from UPP1 and UPP2 measurements in a certain animal. “After Tx/ini” values come from measurements at the terminal experimental point - either 28th day (1 m) or 84th day (3 m). Data before and after injection were analyzed using the non-parametric Wilcoxon test. In groups, where the statistically significant difference was noted the *p* value is presented. Tx, transplantation; ini, injection; MDC, muscle-derived cells; MSC, mesenchymal stem/stromal cells; MDC-MSC, MDCs and MSCs co-transplanted

The general pattern of UPP was similar in all examined animals. The pressure steadily grew in the proximal part of the urethra reaching its peak in the slightly distal section, then it dropped. The representative profiles recorded in animals from three experimental groups at different time points are presented in Fig. 3. The mean MUCP of all animals in the study (*n* = 23) measured at the time of biopsy (UPP1) amounted to 59.2 cm H_2_O (± 12 cm H_2_O) and the mean UPP functional area (FA) at the same time point was 109.2 cm^2^ (± 27.7 cm^2^). There were no significant differences in the mean initial UPP parameters between experimental groups. At the time of injection (UPP2), the mean MUCP in all animals amounted to 59.2 cm H_2_O (± 13.7 cm H_2_O) and mean FA was 109.5 cm^2^ (± 28.0 cm^2^). Again, there were no significant differences in the mean UPP2 parameters between experimental groups. Therefore, for further analyses, in each animal the mean MUCP and FA from UPP1 and UPP2 was calculated and was used as “before Tx” values. The comparison of “before Tx” UPP parameters with “after Tx” UPP parameters was performed for each animal in the study. The individual results are presented in Fig. 4.

The first analysis aimed to evaluate the impact of a transplantation type on urethral closure. If the difference between “before Tx” and “after TX” was less than 5% of “before Tx” it was classified as unaffected. It turned out that only in the co-transplantation group the MUCP and FA measurements after transplantation were higher than before Tx in all six animals. In other groups the results varied. In the MDC group, the MUCP records were increased after Tx in three animals, decreased in one animal and in two animals remained unaffected. The FA values in the MDC group increased in three animals, decreased in two animals and in one animal remained unchanged. In the MSC group, the MUCP value after Tx was increased in three animals and decreased in three animals. The UPP functional area increased in five animals and decreased in one animal. In the PBS group, the MUCP measurement after injection was increased in four animals and remained unaffected in one animal and FA values were increased in four animals and decreased in one animal. Taken together, only in the MDC-MSC group was a statistically significant increase in both UPP parameters noted after transplantation when compared to the pretreatment values (*p* = 0.02 for MUCP and *p* = 0.04 for FA, Wilcoxon test). The second analysis additionally took different time points into account (28 vs 84 days). The mean change in UPP parameters after Tx was calculated for each transplantation type and each time point separately (expressed as change (percentage) in regard to the pre-transplantation values in the same animals, Table 1). At 28 days, the most prominent improvement in both the MUCP and FA parameters was observed in the co-transplantation group (23.2% and 17.8%, respectively, *n* = 3) and almost no change was noticed in the MDC group (2.2% and 1.4% for MUCP and FA, respectively, *n* = 4), despite the fact that DID-derived spots were present in MDC-transplanted urethras at this time point. Interestingly, at 84 days the greatest improvement was observed in the MDC group (32.6% and 50.8% for MUCP and FA, respectively, *n* = 2).

**Table 1:** Proportional changes in urethral pressure profile parameters after transplantation of different cell types

		1 month	3 months
MDC	MUCP	2.2% (±10.3%)	32.6% (±15.8%)
	FA	1.4% (±12.9%)	50.8% (±18.1%)
MSC	MUCP	10.1% (±12.7%)	4.5% (±9.2%)
	FA	11.1% (±16.7%)	23% (±12.3%)
MDC-MSC	MUCP	23.2% (±0.7%)	25% (±5.8%)
	FA	17.8% (±3.0%)	18% (±5.0%)

The values represent mean change (±SEM) 1 month or 3 months after transplantation in comparison to the pre-transplantation values in the same animals. *MUCP* maximal urethral closure pressure, *FA* functional area (of the urethral pressure profile), *MDC* muscle-derived cells, *MSC* mesenchymal stem cells, *MDC-MSC* co-transplantation of MDC and MSC

### The relationship between functional change and other parameters at 28 days

**Fig. 5:**
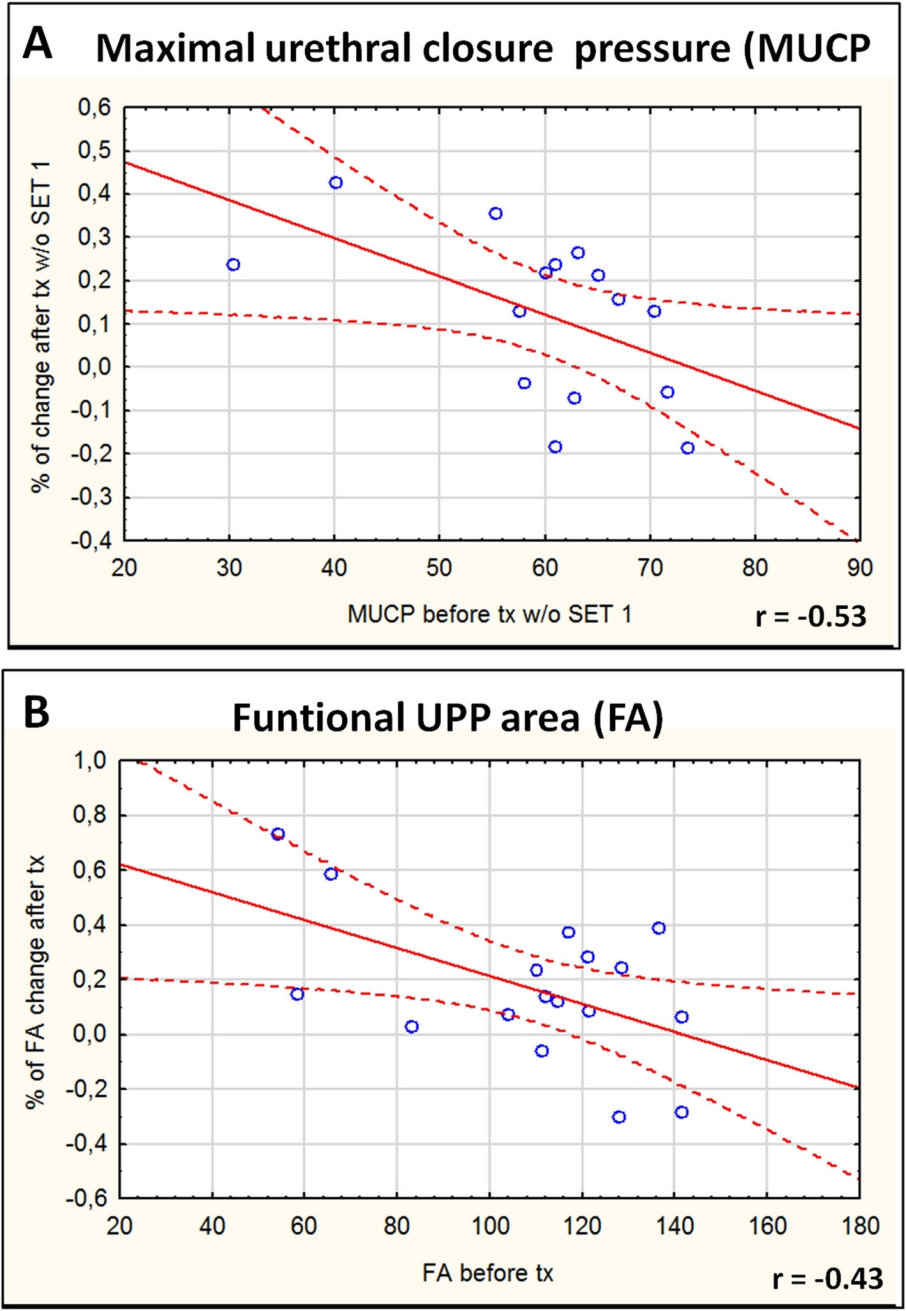
Correlation between the proportional functional changes after transplantation and the initial urethral pressure profile (UPP) parameters. **a** Correlation between maximal urethral closure pressure (MUCP) proportional change and initial MUCP in each transplanted animal; **b** correlation between area under the UPP curve (FA) proportional change and initial FA in each transplanted animal; r, correlation coefficient

As functional change after transplantation procedure varied markedly between animals, additional analyses were performed to verify if more factors (other than the transplantation type) had an impact on the UPP parameters at the terminal time point. Although the mean number of injected cells in the experimental groups did not differ significantly, the number of injected cells in the whole study ranged from 19.8 × 10^6^ to max 36.2 × 10^6^). However, there was no correlation between the number of injected cells (within ranges used) with the proportional MUCP and FA changes after transplantation (*r* = 0.046 and 0.004, respectively, data not shown). Next, the significance of DID-derived spot number in the urethra was analyzed (as we previously reported the limited precision rate of transurethral injections). Again, no correlation was noted between the proportional MUCP and FA changes after transplantation and the number of spots detected (calculated in both the whole urethral wall and only in the external urethral sphincter, which is the desired location) (data not shown). Furthermore, we analyzed if the parameters of initial UPP have an impact on functional change. Strong/moderate negative correlation was noted between the proportional functional changes after transplantation and the initial UPP parameters (*r* = − 0.66 for MUCP, *r* = − 0.53 for FA). The additional analysis of initial UPP parameters revealed that measurements of both MUCP and FA recorded in the first set of the study (one animal from each transplanted group) displayed distinctly lower values than in all remaining sets. This observation suggested that UPP1 parameters from the first set were affected by the learning curve of the experimenters. Therefore, the analysis of correlation was performed once more after removal of data from the 1st set. There was weaker, but still moderate, negative correlation between UPP1 parameters and functional change (*r* = − 0.53 for MUCP and *r* = − 0.43 for FA, Fig. 5).

### The differentiation of transplanted cells in the urethral wall

#### Differentiation of cells injected into the striated muscle

**Fig. 6:**
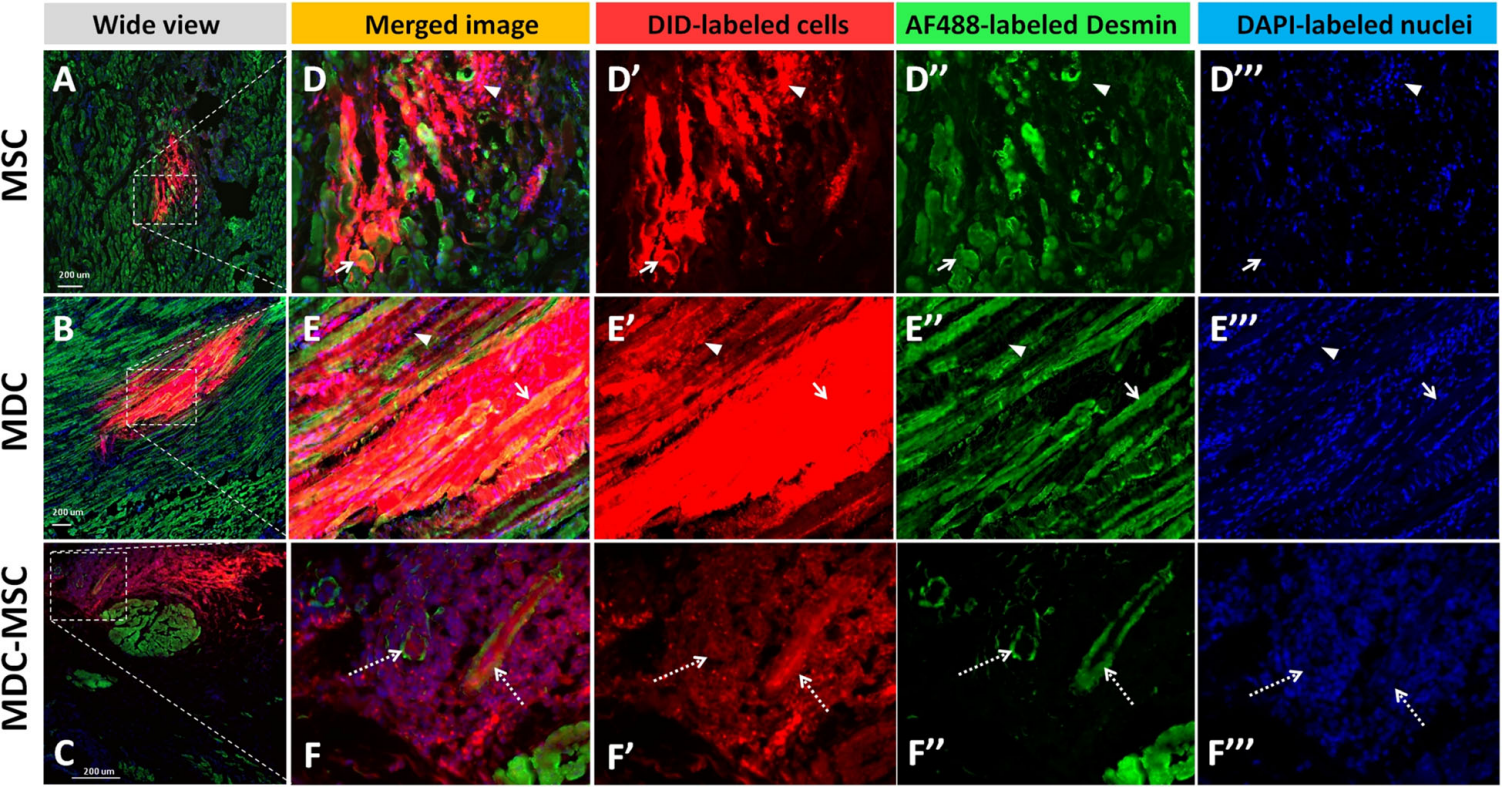
Myogenic differentiation of transplanted cells within the urethral wall (28th day time point). Images of cross-sections after immunohistochemical staining. 1,1′-Dioctadecyl-3,3,3,-tetrametlylindodicarbocyanine-5,5-disulfonic acid (DID)-labeled transplanted cells are red, AF488-labeled desmin is green, 4',6-diamidino-2-phenylindole (DAPI)-labeled nuclei are blue. (A) Urethra from the mesenchymal stem/stromal cells (MSC) group; (B) urethra from the muscle-derived cells (MDC) group; (C) urethra from the co-transplanted group. Scale bar 200 μm; D, E, F images corresponding to the area marked up on the neighboring picture (with merged and separated fluorescent channels). Arrows indicate areas where DID-labeled cells are present within muscle fibers; arrowheads indicate areas where DID-labeled cells remain undifferentiated; pointed arrows (MDC-MSC) indicate DID^pos^/desmin^pos^ elongated structures with centrally located nulcei (de novo created young muscle fibers)

If cell depots were successfully located in the external urethral sphincter (EUS), the presence of striated muscle fiber that displayed DID-derived fluorescence could be identified after transplantation of both cell types - MSCs and MDCs (Fig. 6a, b, d, e). This observation suggests that both MSC and MDC were able to contribute to muscle fiber formation. However, many DID-positive cells within muscle-located depots remained mononuclear and were located between muscle fibers.

#### The fate of cells injected outside the muscle layer

**Fig. 7:**
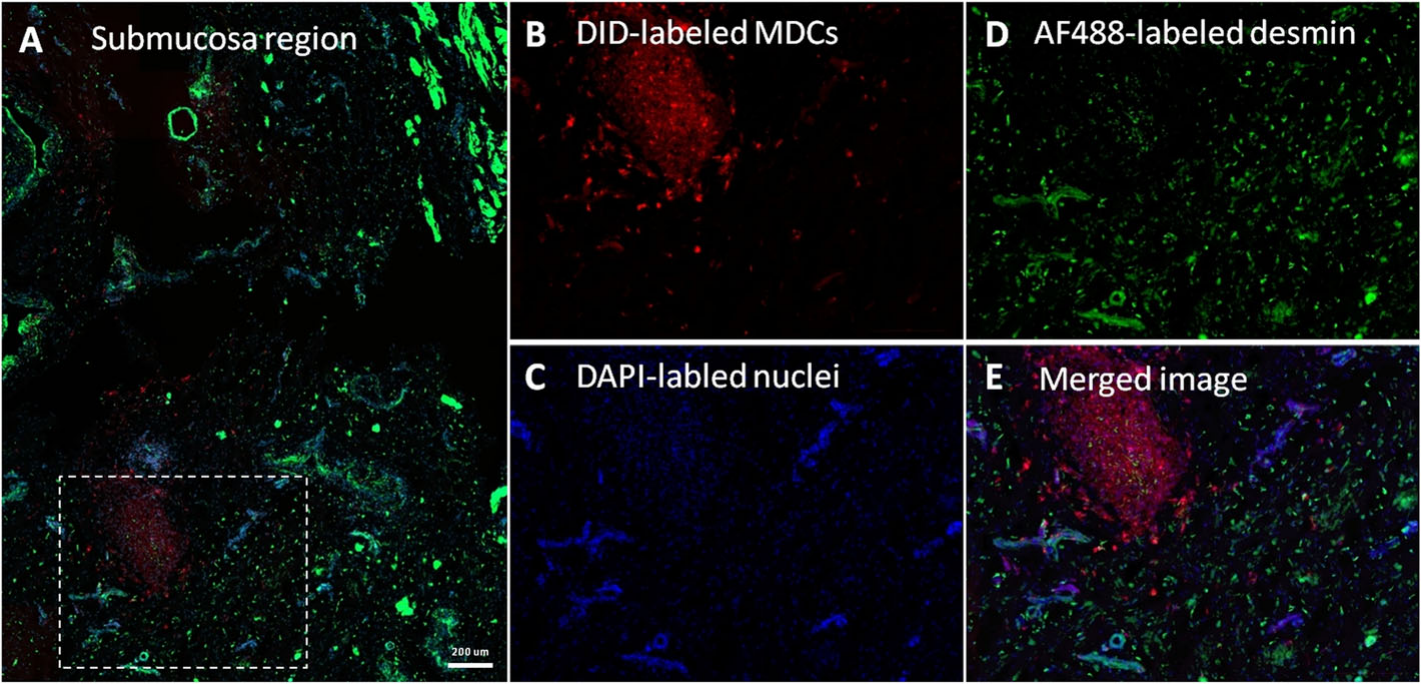
The fate of transplanted muscle-derived cells (MDCs) in the submucosal layer. Images of cross-section after immunohistochemical staining. 1,1′-Dioctadecyl-3,3,3,-tetrametlylindodicarbocyanine-5,5-disulfonic acid (DID)-labeled transplanted MDCs are red, AF488-labled desmin is green, 4',6-diamidino-2-phenylindole (DAPI)-labeled nuclei are blue. **a** Wide view of the submucosal region with a DID-positive depot; (**b**–**e**) Represent the same field of view (corresponding to the area marked on the neighboring picture). Numerous DID^neg^/desmin^pos^ mononulear cells can be observed around the DID^pos^ depot. Scale bar (**a**), 200 μm

**Fig. 8:**
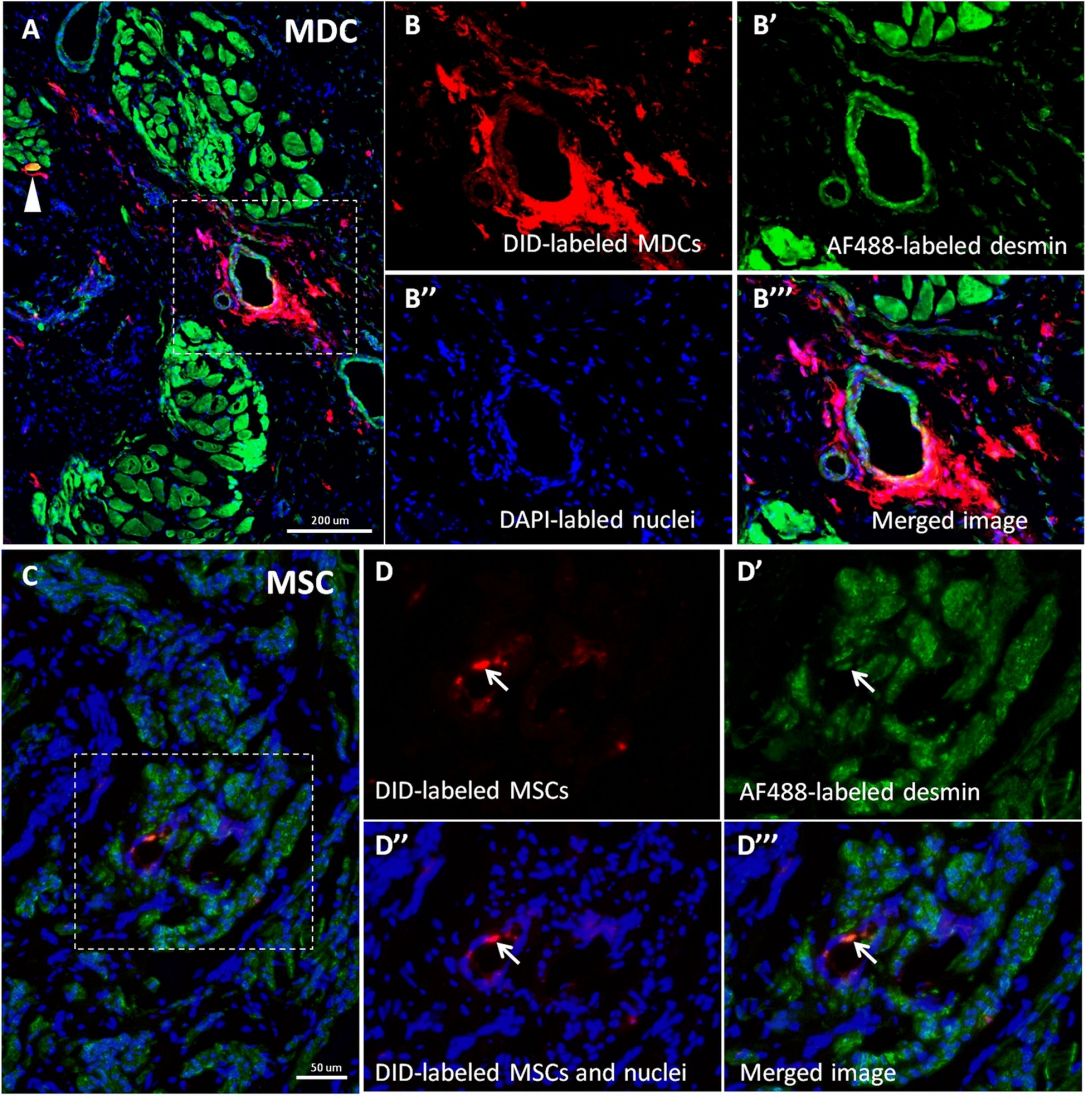
The contribution of transplanted muscle-derived cells (MDCs) and mesenchymal stem/stromal cells (MSCs) to the blood vessel formation. Images of cross-section after immunohistochemical staining. 1,1′-Dioctadecyl-3,3,3,-tetrametlylindodicarbocyanine-5,5-disulfonic acid (DID)-labeled transplanted MDCs are red, AF488-labeled desmin is green, 4',6-diamidino-2-phenylindole (DAPI)-labeled nuclei are blue. (A) urethra from the MDC group. Arrowhead indicates the DID^pos^/desmin^pos^ muscle fiber cross-section. B, B′, B″, B″‘ represent the same field of view (corresponding to the area marked on the neighboring picture). The contribution of DID^pos^ cells to the blood vessel wall formation can be observed. (C) Urethra from the MSC group. D,D’,D″,D″‘ represent the same field of view (corresponding to the area marked on the neighboring picture). The contribution of DID^pos^ cells to the blood vessel wall formation can be observed (marked with the arrow). Scale bars, (A) 200 μm, (C) 50 μm

Only in one goat from the MDC-MSC 28-day group was formation of structures observed that looked like young muscle fibers created de novo (Fig. 5c, f). Those structures were tubular, DID^pos^/desmin^pos^ with nuclei located centrally on a cross-section. This depot was located outside of the EUS, but in close vicinity to the smooth muscle bundle. In the MDC and MSC groups the formation of DID^pos^ muscle structures outside of muscle layers was not observed. The depots located in the submucosal region usually could be recognized as packed clusters of DID^pos^ cells. In the MDC group some of those cells expressed desmin but they remained mononulcear (Fig. 7). In some MDC-injected urethras the depots located in the submucosal region were surrounded by a number of mononuclear, desmin^pos^/DID^neg^ cells (Fig. 7). The scattered DID^pos^ cells were observed in the urethral wall around depots, which suggested the migration of transplanted cells. The number of DID^pos^ cells observed outside depots was low and varied between urethras and also between depots within one urethra. In two cases (one urethra from the MDC group and one from the MSC group) the DID^pos^ cells were identified in the blood vessel wall (Fig. 8) suggesting the possible contribution of transplanted cell to angiogenesis.

## Discussion

In the present study we evaluated the effects of autologous intraurethral transplantation of myogenic cells, mesenchymal stem cells and both cell types in a co-transplantation procedure. The study was performed on large experimental animals in order to enable best mimicking of procedures used in human patients. Previously, our group has demonstrated that the goat can be an appropriate experimental animal for testing intraurethral cell transplantation [18]. We have also optimized the isolation and culture conditions of caprine myoblasts and MSCs [10, 18].

An important issue in testing the efficacy of cell therapy is an animal model of a disease. In regards to the sphincter deficiency, numerous different experimental injury techniques have been proposed [21]. Most of them have been applied in rodents. A comparative study performed by Praud et al. indicated that only longitudinal sphincterectomy causes definitive and irreversible incontinence [22]. In large experimental animals two methods have been described – the urethral distention in pigs [23] and partial sphincterectomy in dogs [24]. Although the urethral distention seems to reflect the changes present in intrinsic sphincter deficiency in women, the durability of the model was assessed only 28 days after injury. As in the present we planned to test the efficacy of cell transplantation also after 3 months, and we decided to use an alternative strategy. Instead of inducing sphincter injury, we have used adult/aged animals after multiple deliveries. We assumed that in those animals the urethral closure can be weakened and therefore it could be improved by the test procedure. Indeed, we have noticed statistically significant improvement in functional urethral parameters in one experimental group (co-transplantation group). The advantage of this approach is that no severely traumatic procedure has to be performed, which limits animal suffering. Moreover, freshly injured tissue constitutes a specific niche for grafted stem/progenitor cells, while the most probable candidates for cell-based therapy are women suffering from stress urinary incontinence (SUI) with no history of recent direct urethral injury. Therefore, we claim that using adult/aged, multiparous females is an attractive alternative to animal models of sphincter injury, especially in large animal species.

One of the most important problems in evaluating cell therapy effectiveness is the diversity of protocols used in different studies. One of those variables is the cell dose. In regards to intraurethral injections in human patients the number of implanted cells differ from 0.6 × 10^6^ to 200 × 10^6^ [25, 26]. The most commonly used cell dose for intraurethral injections in previously reported clinical trials ranges between 25 and 50 × 10^6^ [27]. In the present study the mean number of injected cells were within this range and amounted to 29 × 10^6^. Our previous anatomical evaluation of the female caprine urethra revealed that both the whole organ and the EUS size are similar to those reported in women [18]. Therefore, the cell dose used in this study corresponded well with protocols from the majority of hitherto reported clinical trials. Nevertheless, the results published by Peters et al. [25] and Carr et al. [28] suggest that higher doses (100–200 × 10^6^ per patient) are more effective for stress urinary incontinence (SUI) treatment. This might partially explain why we have not noticed an improvement in UPP parameters in the MDC and MSC groups in the present study.

Another crucial question in the cellular therapy field is which cell type is optimal for a certain application. For intraurethral injections MDCs or MSCs have been most commonly used in both preclinical and clinical studies. In studies on small animal models the functional improvement was observed in all published reports regardless of cell type used [3]. In studies, where autologous intraurethral cell transfer was tested in large experimental animals the results are more diverse. Significant functional improvement was noticed in some studies [29, 30]. In one study the results varied depending on cell dose [31] and finally, in two studies no significant functional improvement was noticed after cell transplantation [32, 33]. Also, in the present study neither MDCs nor MSCs significantly improved the urethral profile parameters. We claim that the explanation of discrepancies between different preclinical studies has at least three components. First - the injection technique, second - the cell dose and third - the pre-transplantation urethral condition. In the present study we injected the cells transurethrally using an endoscope. We have recently demonstrated that the precision of this injection method is limited and can be associated with low efficacy of the procedure. However, this is the technique used in human patients. Second, we have used approximately 30 × 10^6^ of cells per animal. As aforementioned, it is comparable with doses used in human patients, but in the vast majority of preclinical studies performed in rats, dogs and monkeys the relative cell dose is much higher. Finally, we injected cells into non-injured urethra. On one hand we demonstrated that the functional parameters can be significantly improved in non-injured multiparous caprine urethras, but on the other hand our data indicate that the lower initial UPP parameters give the higher chance of improvement after cell transfer. So, it is more difficult to achieve a significant improvement in animals in which the urethra is only slightly affected by multiple deliveries than in animals with acutely injured sphincters.

Although neither MDCs nor MSCs transplanted alone caused significant improvement in total, some interesting observations were made when data from different time points were considered separately. In the MSC group the MUCP and FA were worse at day 84 than at day 28, whereas in the MDC group an opposite trend was observed: the best improvement was noticed in animals from the day 84 time point (Table 1). Similar results were presented by Fu and colleagues. The authors analyzed the effect of intraurethral cell transplantation at the same time points (4 and 12 weeks) and used undifferentiated adipose-derived stem cells and the same cells after myogenic induction. They reported that cells induced into myogenic lineage caused a functionally better effect at the 12-week time point than those that were undifferentiated [34]. In our study, those results additionally corresponded with the DID-derived fluorescent strength - at the 12-week (84-day) time point the mean normalized TRE was markedly higher in the MDC group than in the MSC group. The potential explanation for this observation is that MDCs integrated with the host muscle tissue to a higher extent than MSCs. The integrated cells remain alive and do not proliferate, so the fluorescent signal is preserved. At the same time, functional improvement can be noticed. Indeed, it seemed that MDCs contributed to the muscle fiber formation more often than MSCs, although the histological data were not quantified in the present study. The ability of MDCs to differentiate into EUS muscle fibers was previously undoubtedly demonstrated in other reports [5]. In the case of MSCs, the analogous convincing proof for myogenic differentiation within the EUS has not been previously provided. Presented herein data indicate that transplanted MSCs are able to fuse with muscle fibers of the urethral striated sphincter, but those events are rare and the majority of injected MSCs remained undifferentiated. This can be a potential explanation of a weak functional result at the 12-week time point in the MSC group. However, the restricted number of animals used in this study limits the significance of this assumption. If cells were injected out of the muscle layer, both MSCs and MDCs remained undifferentiated, which confirms our previous results obtained in the porcine model [32]. In the present study we additionally observed that in some (not all) submucosally injected MDC depots the presence of numerous scattered mononuclear, desmin^pos^/DID^neg^ cells was noticed (Fig. 7). These cells were either migrating grafted cells, which underwent divisions and lost DID fluorochrome but remained desmin^pos^ or they are endogenous myogenic progenitors attracted by transplanted cells. As such a phenomenon was not observed around the MSC depots we suppose the first explanation is more probable.

The main objective of the study was to evaluate the effect of MDC-MSC co-transplantation. We hypothesized that MDCs can improve the myogenic activity of MSCs and on the other hand, MSCs can improve the survival of MDCs (by a paracrine effect). Therefore, we assumed that co-transplantation efficacy could be higher than transfer of a single cell-type. The evaluation of MSC myogenic differentiation in the co-transplanted group failed because of the labeling method. For co-transplantation, MSCs were stained with green PKH67 cell membrane fluorophore. Unfortunately, the weak PKH67-derived signal and high tissue autofluorescence prevented the reliable analysis of grafted cells in the urethral sections. This observation confirmed previous reports suggesting that markers with green fluorescence are not optimal for cell tracking after transplantation [35, 36]. In contrast to green dye, the DID-derived fluorescence could be successfully assessed both in explants using the in vivo imaging system and by microscopic evaluation of urethral cross-sections. The DID dye was used for staining cells in the MDC and MSC groups and for staining MDCs in the co-transplanted group. We have used the previously described protocol of ex vivo tissue analysis, which allows the localization of grafted spots within the tissue and enables semi-quantitative estimation of fluorescence strength in the whole organ [20]. The assessment revealed that the median TRE value normalized to injected DID-stained cell number in 28-day urethras was almost twice as high in the co-transplantation group than in the MDC group (6.4E + 09 vs 3.4E + 09). This suggests that co-injection of MSCs improved MDC survival at this time point. This effect was no longer observed 12 weeks after transplantation - at this time point the DID-derived signal was higher in the MDC group than in the co-transplanted group. The decline of membrane fluorochrome-derived fluorescence can result either from labeled cell death or proliferation of labeled cells. However, as functional improvement at day 28 and 84 in the MDC-MSC group was similar, the more probable reason is the gradual graft loss. Nevertheless, the overall functional effect was most prominent in co-transplanted animals as only in this group was the statistically significant improvement noticed in both analyzed functional parameters. Previously, similar results were published by Williams and colleagues [14]. The authors reported that the effect of co-transplantation of cardiac progenitors and MSCs was more effective in the myocardial infarction model in pigs than either cell therapy alone.

It is not clear which mechanism stands behind enhanced efficacy of MDC-MSC co-transplantation. In one co-transplanted urethra we have observed a formation of structures which resembled young myofibers created de novo. Those structures were out of the striated muscle layer. Similar DID^pos^ young muscle fibers were not found in the MDC group. Therefore, we suppose that as the presence of MSCs could encourage MDC to terminal differentiation and enabled formulation of new muscle fibers. In our previous report we have shown that MSC-derived soluble factors increased the fusion index of cultured myoblasts, which supports this hypothesis [10].

## Conclusion

In conclusion, the results presented indicate that adult/aged multiparous goats can be an attractive alternative to the animal models of sphincter injury, especially in large animal species. The myogenic differentiation of MDCs and MSCs transplanted alone into the urethral wall occurs, but only if the cell depot is administered into the EUS. In the case of MSCs, the direct contribution to muscle regeneration is a very rare phenomenon. The results suggest that MDC-MSC co-transplantation can enhance de novo muscle fiber formation and provide greater chance of functional improvement than injection of either MDCs or MSCs alone. Finally, the data presented suggest that the graft capacity declines with time and at the 84th day after transplantation is barely detectable.

## Abbreviations

ANOVA: Analysis of variance
BM: Bone marrow
CellF: Cell number factor to adjust the number of 1,1′-Dioctadecyl-3,3,3,-tetrametlylindodicarbocyanine-5,5-disulfonic acid-labeled cells
DAPI: 4',6-Diamidino-2-phenylindole
DID: 1,1′-Dioctadecyl-3,3,3,-tetrametlylindodicarbocyanine-5,5-disulfonic acid
DMEM: Dulbecco’s modified Eagle’s medium
Em: Emission wavelength
EUS: External urethral sphincter
Ex: Excitation wavelength
FA: Functional area (the area under the urethral pressure profile curve)
FCS: Fetal calf serum
i.m.: Intramuscular
i.v.: Intravenous
IVIS: In Vivo Imaging System
MDC-MSC: Muscle-derived cells - mesenchymal stem cells co-transplantation
MDCs: Muscle-derived cells
MSCs: Mesenchymal stem/stromal cells
MUCP: Maximal urethral closure pressure
ns: Not significant
nTRE: Normalized total radiant efficiency
PBS: Phosphate-buffered saline
ROI: Region of interest
RT: Room temperature
SUI: Stress urinary incontinence
UPP: Urethral pressure profile
